# Drought Increases Consumer Pressure on Oyster Reefs in Florida, USA

**DOI:** 10.1371/journal.pone.0125095

**Published:** 2015-08-14

**Authors:** Hanna G. Garland, David L. Kimbro

**Affiliations:** 1 Department of Biological Science, Florida State University, Tallahassee, Florida, United States of America; 2 Department of Marine and Environmental Sciences, Northeastern University, Marine Science Center, Nahant, Massachusetts, United States of America; Texas A&M University at Galveston, UNITED STATES

## Abstract

Coastal economies and ecosystems have historically depended on oyster reefs, but this habitat has declined globally by 85% because of anthropogenic activities. In a Florida estuary, we investigated the cause of newly reported losses of oysters. We found that the oyster reefs have deteriorated from north to south and that this deterioration was positively correlated with the abundance of carnivorous conchs and water salinity. In experiments across these gradients, oysters survived regardless of salinity if conchs were excluded. After determining that conchs were the proximal cause of oyster loss, we tested whether elevated water salinity was linked to conch abundance either by increasing conch growth and survivorship or by decreasing the abundance of a predator of conchs. In field experiments across a salinity gradient, we failed to detect spatial variation in predation on conchs or in conch growth and survivorship. A laboratory experiment, however, demonstrated the role of salinity by showing that conch larvae failed to survive at low salinities. Because this estuary’s salinity increased in 2006 in response to reduced inputs of freshwater, we concluded that the ultimate cause of oyster decline was an increase in salinity. According to records from 2002 to 2012, oyster harvests have remained steady in the northernmost estuaries of this ecoregion (characterized by high reef biomass, low salinity, and low conch abundance) but have declined in the southernmost estuaries (characterized by lower reef biomass, increases in salinity, and increases in conch abundance). Oyster conservation in this ecoregion, which is probably one of the few that still support viable oyster populations, may be undermined by drought-induced increases in salinity causing an increased abundance of carnivorous conchs.

## Introduction

The community and ecosystem dynamics of natural systems are often defined by the architecture and functional ecology of dominant “foundation” species such as coral and trees [1,2]. The abundance and persistence of these foundation species are, in turn, influenced by a subtle balance between environmental gradients and species interactions [3]. For example, with increasing elevation and environmental stress, dominant alpine plant communities change in both plant species composition and biotic interactions as competitive interactions shift to facilitation [4]. Unfortunately, the conditions and mechanisms that promote persistence of foundation species are increasingly disrupted by anthropogenic disturbances [2,5].

Abundant populations of ungulates, for instance, have depleted hardwood trees throughout the western U.S. [6]. Similarly, an abundant herbivorous crab is expanding salt marsh losses in New England [7]. Because these examples of enhanced consumer pressure coincide with the suppression of top-level predators, it is reasonable to infer that foundation species decline when apex predators are removed by human activities [8]. In addition to the inadequate regulation by top predators, it is clear that foundation species are being affected by environmental change in general and climate change in particular. Increasing anomalies of warm temperature, for example, cause large-scale losses of coral reefs and aspen forests by promoting outbreaks of disease [9] and hydraulic root failure [10].

While environmental change and excessive consumer pressure can independently cause habitat losses, these two stressors can also interactively decrease foundation species. An example concerns Australian coral reefs, where increased precipitation and consequently nutrient-rich runoff support blooms of coastal phytoplankton, which in turn increase the growth rate of planktotrophic starfish larvae (*Acanthaster planci*). By reducing the amount of time that the larvae remain small and therefore vulnerable to predators, precipitation- and runoff-induced blooms of phytoplankton may explain starfish outbreaks that deplete the reefs [11]. Across ecosystems, a variety of predicted environmental changes and anthropogenic stressors could independently or interactively harm foundation species [12,13]. Thus, the empirical testing of when, where, and how multiple factors reduce the persistence of foundation species is fundamental to the conservation and restoration of key habitats.

For centuries, coastal ecosystems depended on the services provided by oysters. The benefits of oyster reef habitat include the enhancement of commercially important invertebrates and finfish, stabilization of shorelines, filtration of coastal water, and removal of excess nitrogen [14,15]. As a result of habitat degradation, overharvesting, and eutrophication, the global abundance of this habitat has declined by 85% [16]. Most of the world’s remaining reefs are concentrated in six ecoregions, and five of these are located in the United States. Because the increasing number of people that live along coasts negatively influences oysters and their services [17], understanding and mitigating further change in these ecoregions is a key conservation goal.

**Fig 1:**
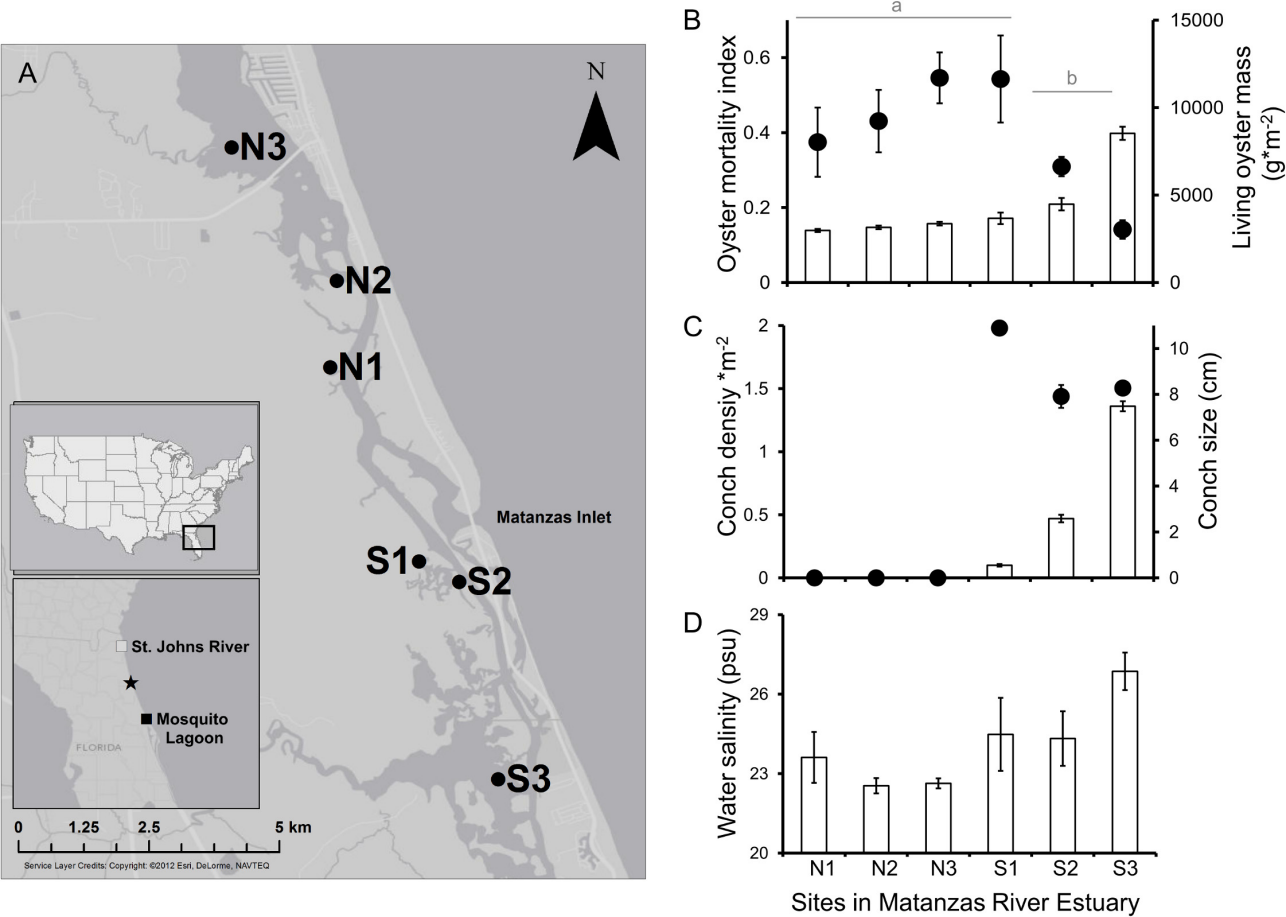
Variation in oyster reefs, crown conchs, and water salinity across study sites in the Matanzas River Estuary (MRE). (A) Map of six study locations in the MRE with the first inset illustrating the Floridian ecoregion, and the second inset illustrating the location of the MRE (star symbol) within this ecoregion. (B) The mortality index (open bars, primary Y-axis) and living biomass of adult oysters (closed circles, secondary Y-axis) on oyster reefs throughout the MRE; different letters above the horizontal lines denote significant differences based on Scheffe’s post-hoc test (p < 0.05). (C) Crown conch abundance (open bars, primary Y-axis) and size (closed circles, secondary Y-axis) on oyster reefs throughout the MRE. (D) Salinity of water across sites in the MRE. All maps were produced in ArcGIS by E. Pettis.

In one of the six ecoregions that support commercial harvesting of oysters (Floridian, Fig 1A, [16]), oyster (*Crassostrea virginica*) abundance may be declining. According to stakeholders (S1 Table) of an estuary in this ecoregion (Matanzas River Estuary, hereafter MRE; Fig 1A), many commercial oyster leases were abandoned in 2008 because oysters consistently died before reaching market size. Assuming that this commercial failure represents a true decline in oyster reefs, understanding its causes will be difficult because a number of changes have recently occurred in this system. Just before the purported oyster decline, stakeholders observed an increase in the crown conch (*Melongena corona*), which is a known consumer of oysters [18]. Although the crown conch may be depleting oysters as a result of reduced regulation from its predators including the stone crab (*Menippe mercenaria*, [19]) and the horse conch (*Triplofusus giganteus* [20], oyster reefs may be declining because of a prolonged regional drought in the southeast U.S. [21], which can alter water salinity and temperature in ways that increase disease [22,23] and desiccation stress [24]. Given that consumer-induced mortality of oysters has been linked to increased water salinity [25], oyster declines may also be due to an interaction between estuarine salinization and consumer pressure. However, the extent and causes of oyster loss in this ecoregion remain unknown.

Here, we used a comparative-experimental approach to identify both the proximal and ultimate causes for the putative decline in oyster numbers within the MRE. Our study had four parts. First, we monitored the MRE to establish correlative relationships among oyster reefs, environmental factors, and trophic structure. Second, along a potentially important environmental gradient of water salinity, we conducted experiments that established the spatial variation in conch abundance as the proximal cause for variability in reef conditions. Third, we used a field-tethering experiment, monitoring, and the re-analysis of a published laboratory experiment [26] to identify a drought-induced increase in water salinity as the ultimate cause of oyster decline. Finally, we examined time series and oyster reefs throughout two other estuaries in this ecoregion to determine whether the overconsumption of reefs by conchs is occurring regionally.

## Methods

### Ethics statement

The name of the authority that issued permission to conduct research at sites in St. Johns River estuary was the National Park Service Timucuan Ecological Historic Preserve. The name of the authority that issued permission to conduct research at sites in the Matanzas River estuary was the Guana Tolomato Matanzas National Estuarine Research Reserve (GTM NERR). The name of the authority that issued permission to conduct research at sites in Mosquito Lagoon (Brevard county) was the National Park Service Cape Canaveral National Seashore. To conduct research at all of the locations, permission was granted by the Florida Fish and Wildlife Conservation Commission. This research involved observing invertebrate animals in the natural environment and using the same invertebrate animals in experiments within the natural environment. Therefore, we were not required to seek approval from the Institutional Animal Care and Use Committees at Florida State University and Northeastern University. All animals were treated humanely and all surviving animals were released to their origin locations at the conclusion of the experiment.

### Study system

Most of this research was conducted in the GTM NERR, which is located in the MRE (29.91386°N, 81.28368°W). Shorelines north of the Matanzas inlet are dominated by intertidal oyster reefs that border salt marsh (*Spartina alterniflora*), and shorelines south of the Matanzas inlet are dominated by oyster reefs that border salt marsh and mangrove (*Avicennia germinans*). The estuary’s hydrodynamics have been well described [27]: areas closer to the inlet are influenced by tidal excursion, and areas farther from the inlet are influenced by freshwater input. The consumer of focus was the crown conch, which is a direct-developing prosobranch gastropod with a generalist diet that occurs intertidally along the Gulf and Atlantic coasts of Florida [28].

### MRE monitoring

Oyster reefs south of the Matanzas inlet historically supported commercial harvesting. In recent years, however, oysters in this area have died before reaching market size. We surveyed six sites along 13 km of estuary that were suspected to span locations with and without market size oysters (Fig 1A). At each site, we randomly selected four oyster reefs separated by 100 m and extracted all material within six spatially stratified quadrats per reef (quadrat = 0.0625 m^2^). In the laboratory, we counted juvenile (< 25 mm) and adult (> 25 mm) living oysters as well as adult oysters that were newly “gaping”; such gaping adults have two intact shells but lack oyster tissue and sessile invertebrates within the internal shell cavity, which indicate recent mortality due to stress or a consumer that does not damage the shell such as the conch. We also measured adult oyster shell length and quantified the abundance of adult mud crabs (*Xanthidae;* carapace width ≥ 12 mm) and Atlantic oyster drills (*Urosalpinx cinerea*). While the former species consumes juvenile oysters [29], the latter species consumes juvenile [30] and adult oysters [31]. In addition, we quantified the number and size of conchs along a 10.0-m transect (width = 1.0 m) paralleling the shore. These variables were surveyed in June of 2011 and 2012. We did not quantify other consumers of oysters such as the blue crab (*Callinects sapidus*) and the stone crab (*Menippe mercenaria*) because the foraging activity of these consumers destroys oyster shells. Meanwhile, the field pattern of oyster reefs that motivated our study consisted of large adult oysters with two intact valves (Fig 1B).

In June, July, August, and December of 2011, we collected point samples of water temperature, salinity (measured on the practical salinity scale, psu), and dissolved oxygen with a hand-held YSI meter (model 556). In the summer of 2011, we quantified the availability of phytoplankton (chlorophyll *a*) for oysters once per month via triplicate water samples (250 ml, 0.5 m depth) and standardized laboratory methods [32]. During this period, we also monitored submergence time of reefs and aerial temperature during low tides with an Onset HOBO logger (model U20-001-04). Because salinity was strongly correlated with the mortality index of oyster reefs, subsequent monitoring focused on salinity. From January to September 2012, we used an Onset HOBO conductivity logger (model no: U24-002) to record salinity and water temperature at 30-minute intervals.

### Proximal causal factor

In June 2011, we randomly selected one reef at each of the six survey sites and established 12 experimental units (1.0-m interval) at the mid-point of the oysters’ intertidal distribution. Experimental units were randomly assigned among three treatments: exclosure, exclosure-control, and control (no exclosure). The exclosures and exclosure-controls (0.3 × 0.3 × 0.5 m) were constructed of vinyl-coated wire mesh (12 × 12 mm), but two mesh walls were removed from the exclosure-control to mimic material effects without restricting consumers. We collected live oyster clusters (biomass 100–250 g) from a common location within the MRE, and placed two clusters in each experimental unit. In this experiment, which was designated experiment 1, we quantified live and gaping oysters as well as oyster consumers in each experimental unit every 2 weeks for 2.5 months.

In June 2012, we conducted a similar experiment, which was designated experiment 2, with juvenile oysters to verify that survivorship at this life stage did not account for spatial variation in reef condition. Experiment 2 was conducted at one site north and one site south of the Matanzas inlet (N2, S3; Fig 1A), which sufficiently represented the spatial variation in reef biomass, gaping oysters, salinity, and conch abundance detected in our surveys (Fig 1). At each site, we established three 18.0-m transects parallel to the shore and installed 36 experimental units at 1.0-m intervals along each transect. Experimental units contained 12 juvenile oysters (mean length 8 mm) attached to a vertically oriented ceramic tile (13 × 13 cm) with marine epoxy. Oysters of the same origin, age, and size were obtained from a hatchery. These experimental units were randomly assigned among the same three treatments listed above. Every 2 weeks for 3.5 months, we quantified juvenile oyster survivorship.

### Ultimate causal factor(s)

Because our experiments 1 and 2 identified conch abundance as the proximal cause for reef deterioration, our subsequent research focused on the controls of conch abundance. First, we conducted a tethering experiment, which was designated experiment 3, at three sites (S1–S3) using three size classes of conchs: large (mean length 108 mm), medium (mean length 80 mm), and small (mean length 68 mm). To avoid expanding the conch’s spatial distribution, we excluded sites without conchs (i.e., N1–N3) from this experiment. This was considered acceptable because sites S1–S3 represented a strong gradient in conch abundance, with S1 mirroring the northern sites (Fig 1B). On five reefs at each site and along the mean low tide line, we installed five tethered conchs of each size class at 2.0-m intervals. We used marine epoxy to adhere a fishing swivel to the shell of each conch, and we tied a tether line (100-lb-test monofilament, 1.0 m length) to the swivel. Each conch was attached by its tether to a vertical PVC pipe extending 0.1 m from the benthos. To test for tethering malfunctions, we placed three conchs in separate enclosures (0.13 × 0.13 × 0.18 m) and on each reef. During the experiment, we did not observe tether failure. Conch status (live, dead, or missing) was recorded weekly from August through November 2012 (3 months). Conch survivorship was assessed again in August 2013 (12 months). On one reef at each site, we also tested for spatial differences in conch growth by enclosing five juvenile conchs (mean length 69 mm) in separate cages with an oyster cluster (biomass 100–250 g) for food. Between September and November 2012, we monitored changes in conch shell length.

In the tethering experiment, all conchs survived and thus a spatial gradient in predation pressure failed to explain the spatial gradient in conch abundance. Although our surveys demonstrated that low water salinity may limit the distribution of conchs, the survivorship of tethered adult and sub-adult conchs was equally high among sites and thus across a salinity gradient. However, our experiments did not address whether low salinity influenced conch reproduction. To address this subject, we formally analyzed the descriptive results of a previously published experiment [26]. This experiment, which was designated experiment 4, subjected encapsulated conch larvae to one of nine salinity levels ranging from 8 to 32.8 psu. These investigators destructively sampled larvae to determine their status (live or dead) after 7 and 14 days of exposure, but they did not evaluate the functional relationship between water salinity and larval mortality. See [26] for full details of this experiment.

Since 2002, GTM NERR has recorded salinity in Pellicer Creek, which flows into the portion of the MRE with conchs (29.66707°N, 81.25740°W). We calculated seasonal salinity averages between 2002 and 2012. The residence time and salinity of water in this area are most influenced by discharge from Pellicer Creek and precipitation [27]. As a result, we used the creek’s daily discharge rate (monitored by the United States Geological Survey) and local precipitation data to generate seasonal averages of creek discharge and precipitation.

### Regional trends

The catch per unit effort (CPUE, landings/trips) of oysters is monitored by the Florida Fish and Wildlife Conservation Commission (http://myfwc.com/research/saltwater/fishstats/commercial-fisheries/landings-in-florida/) in one estuary that is north of the MRE (St. Johns River estuary, St. Johns County) and another that is south of the MRE (Mosquito River Lagoon, Brevard County). These are the two major estuaries of the ecoregion, and they are the only two for which oyster CPUE is recorded. In May 2012, we partitioned the length of each estuary into five zones. In each zone, we randomly surveyed five oyster reefs (n = 25 per estuary) and extracted all contents within one quadrat per reef (area = 0.0625 m^2^) and processed each quadrat as described in *MRE Monitoring*. In each zone, we monitored water temperature and salinity with a hand-held YSI meter. We also quantified the number of conchs along a 10.0-m transect paralleling the shore. Because of similarities in reef condition and conchs between the MRE and the Mosquito River Lagoon, we obtained and used a salinity time series for the latter estuary (http://floridaswater.com/watershedfacts/factPages/02248000.html). These data were used to generate seasonal averages in salinity that matched the salinity time series of Pellicer Creek in the MRE (i.e., 2002–2012).

### Statistical analyses

#### MRE monitoring

We used a one-way analysis of variance (ANOVA) with site as a fixed factor to test whether living oyster biomass, oyster mortality index, and adult oyster length differed among sites. Because all ANOVAs were significant, we then used three separate Scheffe’s post-hoc tests to evaluate whether each response variable differed between sites with (S2-S3) and without (N1-N3, S1) reports of oyster losses. Adult oyster density and lengths were converted into biomass via a length–biomass index (y = 0.63x – 15.99, R^2^ = 0.80), which was developed by processing 900 oysters of varying shell lengths in the laboratory. For each quadrat, we calculated a mortality index of adult oysters as the ratio of gaping oysters to total oysters (live + gaping).

We used a model-selection approach to identify the best explanation for the spatial variability in the biomass and mortality index of oysters. For 2 years, we monitored a variety of potentially important biotic (Table 1) and abiotic (Table 2) factors. Before proceeding, we collapsed the data to standardize for unequal temporal and spatial replication among variables: we generated annual means from monthly values and then averaged the annual means of each site into one value. Candidate linear models included a null model (intercept equal to 1) and all possible single-factor models. We used Akaike’s Information Criterion corrected for small sample size (AIC_c_) to identify the most parsimonious model [33]. For both the biomass of living oysters and the mortality index of oyster reefs, we used linear regression to evaluate its correlation with the strongest explanatory variable.

**Table 1:** Summary of biotic factors on oyster reefs of the MRE.

Site	Living oyster biomass (g)	Adult oyster mortality index	Adult oyster length (mm)	Crown conch density (m^2^)	Crown conch length (cm)	Mud crab density (m^2^)
N3	8028.23 ± 1979.6	0.13 ± 0.004	54.79 ± 3.1	0 ± 0	0 ± 0	11.3 ± 2.9
N2	9231.41 ± 1784.8	0.14 ± 0.005	55.27 ± 1.8	0 ± 0	0 ± 0	1.4 ± 1.0
N1	11702.2 ± 1456.9	0.16 ± 0.01	56.94 ± 1.2	0 ± 0	0 ± 0	5.2 ± 4.2
S1	11635.25 ± 2489.1	0.17 ± 0.02	52.52 ± 1.9	0.10 ± 0.02	10.9 ± 0.2	6.2 ± 2.1
S2	6632.81 ± 553.5	0.21 ± 0.02	45.85 ± 0.8	0.47 ± 0.06	7.9 ± 0.2	5.3 ± 2.2
S3	3032.16 ± 530.7	0.40 ± 0.02	39.75 ± 0.5	1.36 ± 0.08	8.3 ± 0.1	17.4 ± 11.5

Values in cells represent mean ± standard error.

**Table 2:** Summary of abiotic conditions on oyster reefs in the MRE.

Site	Water temp (°C)	Water salinity (psu)	Dissolved oxygen (mg/L)	Chl *a* (μg/L)	Proportion of day that reef is submerged	Maximum air temp (°C)
N3	29.07 ± 0.5	23.61 ± 0.96	4.40 ± 1.5	10.32 ± 1.2	0.52 ± 0.02	40.14 ± 4.8
N2	27.86 ± 0.01	22.54 ± 0.29	6.11 ± 0.9	4.78 ± 0.2	0.49 ± 0.02	36.85 ± 1.8
N1	26.66 ± 0.04	23.63 ± 0.19	4.95 ± 1.1	4.96 ± 1.1	0.46 ± 0.02	43.61 ± 3.9
S1	27.21 ± 0.02	24.48 ± 1.38	4.56 ± 0.2	9.62 ± 1.1	0.18 ± 0.01	39.12 ± 4.9
S2	27.87 ± 0.1	24.32 ± 1.03	2.97 ± 0.7	15.35 ± 1.8	0.53 ± 0.02	38.44 ± 2.8
S3	28.87 ± 0.6	26.86 ± 0.71	5.33 ± 0.4	7.84 ± 0.4	0.53 ± 0.02	36.2 ± 1.4

Values in cells represent mean ± standard error.

#### Proximal causal factor

For the field experiment on adult oyster survivorship (experiment 1), we used a two-way ANOVA with site and treatment as fixed factors. For the juvenile oyster experiment (experiment 2), which only used two sites, we designated site and treatment as random and fixed effects, respectively. For both experiments, survivorship was analyzed after 2 weeks because prey were depleted in control treatments beyond this time. The end-point results from exclosure treatments were analyzed using a one-way ANOVA with site as a fixed effect in experiment 1 and with site as a random effect in experiment 2. Before conducting ANOVAs, we logit transformed proportional survivorships and verified that variances were homogeneous.

Because results suggested that consumption of oysters increased southward, we calculated an effect-size for consumer pressure at each site. Replicate effect sizes for each site were calculated by subtracting each replicate of the exclosure treatment from the average survivorship of all control replicates at that site. We conducted a linear regression between the strength of consumer pressure on oysters (i.e., effect size) as a function of conch abundance from our surveys. This regression was repeated using conch abundance in our experimental units as the predictor.

#### Ultimate causal factor(s)

For the tethering experiment (experiment 3), we used a two-way ANOVA to test for the effect of site, size class, and their interaction on conch survivorship after 3 months. This analysis was repeated for survivorship after 12 months. To test for site differences in conch growth, we used a one-way ANOVA with site as a fixed factor. Because spatial differences in the growth and survivorship of conchs were not strong, we focused on the abiotic factor that best explained spatial variation in the oyster mortality index. As a result, we used linear regression to evaluate the relationship between conch abundance and water salinity. For the laboratory experiment (experiment 4) conducted by [26], we used a logistic regression to evaluate the relationship between the probability of larval mortality and water salinity after 14 days of exposure. We did not consider data from the 7-day evaluation, because survivorship was 100% at all salinities. After identifying salinity as the most likely control of conchs, we used a multiple linear regression to evaluate the effect of precipitation and discharge on salinity. Because only discharge was significant, we used separate regressions to examine whether salinity and then discharge changed between 2002 and 2011. Finally, we used regression to evaluate whether freshwater discharge from Pellicer Creek was related to precipitation.

#### Regional trends

Regression was used to evaluate whether annual CPUE changed over the last decade in the St. Johns River and Mosquito River Lagoon. We then used separate ANOVAs to evaluate whether the following three factors currently differ between these two estuaries: live oyster biomass, conch density, and water salinity. For these analyses, we considered reef as a replicate value for oysters and conchs (n = 25/estuary) and zone as a replicate for salinity (n = 5/estuary). Because the Mosquito River Lagoon displayed declining CPUE, high conch abundance, and relatively lower biomass of oysters, we used regression to evaluate whether its water salinity increased over the last decade. Water-monitoring locations were not standardized within each estuary. Therefore, we normalized the time series by subtracting each annual mean from the estuary’s decadal mean and then dividing these differences by the estuary’s decadal standard deviation.

## Results

### MRE monitoring

In comparison to sites N1, N2, N3, and S1, the two southernmost sites of the MRE (sites S2 and S3) had significantly lower oyster biomass (Scheffe-Q 21267.19 > F_3,18, α = 0.05_ 17194.65), smaller oysters (Scheffe-Q 48.32 > F_3,18, α = 0.05_ 18.75), and greater indices of oyster mortality (Scheffe-Q 0.62 > F_3,18, α = 0.05_ 0.34; Fig 1B and Table 3). Of several potential causal variables (Tables 1–2), conch abundance was identified as the strongest predictor of oyster biomass and mortality index (biomass: w = 0.51, ∆AIC_c_ = 0.0, S2 Table; index: w = 0.99, ∆AIC_c_ = 0.0; S3 Table), with the latter metric increasing from north to south in the MRE (Fig 1C). Although high water salinity (Fig 1D) was associated with a high index of oyster mortality and was identified as the second strongest explanatory factor, the highest ranked model with conchs was 198 times more powerful (salinity model: w = 0.005, ∆AIC_c_ = 10.6).

**Table 3:** Analysis of Variance (ANOVA) results of surveys and experiments within the MRE. In the survey, the effect of site on (a) oyster biomass, (b) oyster mortality index, and (c) adult oyster length.

Research objective	Response variable	Source	df	Sum Squares	Mean Square Error	F-ratio	P-value
Field survey	(a) Oyster biomass	Site	2	1.19	0.59	16.48	<0.001*
		Error	22	0.80	0.036		
Field survey	(b) Mortality index	Site	1	0.40	0.40	39.27	<0.001*
		Error	22	0.22	0.01		
Field survey	(c) Adult oyster length	Site	1	778.86	778.86	50.2	<0.001*
		Error	22	341.36	15.52		
Field experiment 1	(d) Adult oyster survivorship	Site	5	60.17	12.03	51.61	<0.001*
		Treatment	2	20.16	10.08	43.24	<0.001*
		Site x Treatment	10	23.52	2.35	10.09	<0.001*
		Error	120	27.98	0.23		
Field experiment 1	(e) Adult oyster survivorship	Site	5	0.03	0.006	1.37	0.28
		Error	17	0.08	0.005		
Field experiment 2	(f) Juvenile oyster survivorship	Site	2	249.05	124.53	428.28	<0.001*
		Treatment	1	36.72	36.72	126.3	<0.001*
		Site x Treatment	2	31.6	15.8	54.34	<0.001*
		Error	210	61.06	0.29		
Field experiment 2	(g) Juvenile oyster survivorship	Site	1	0.19	0.19	0.53	0.47
		Error	70	25.30	0.36		

In experiment 1, (d) the effects of site and treatment on adult oyster survivorship over 2 weeks and (e) the effect of site on *adult* survivorship within cage treatments after 2.5 months. In field experiment 2, (f) the effects of site and treatment on *juvenile* oyster survivorship over 2 weeks as well as the effect of site on *juvenile* survivorship within cage treatments after 3 months. Asterisks denote ANOVAs with significant results (p < 0.05).

### Proximal cause

**Fig 2:**
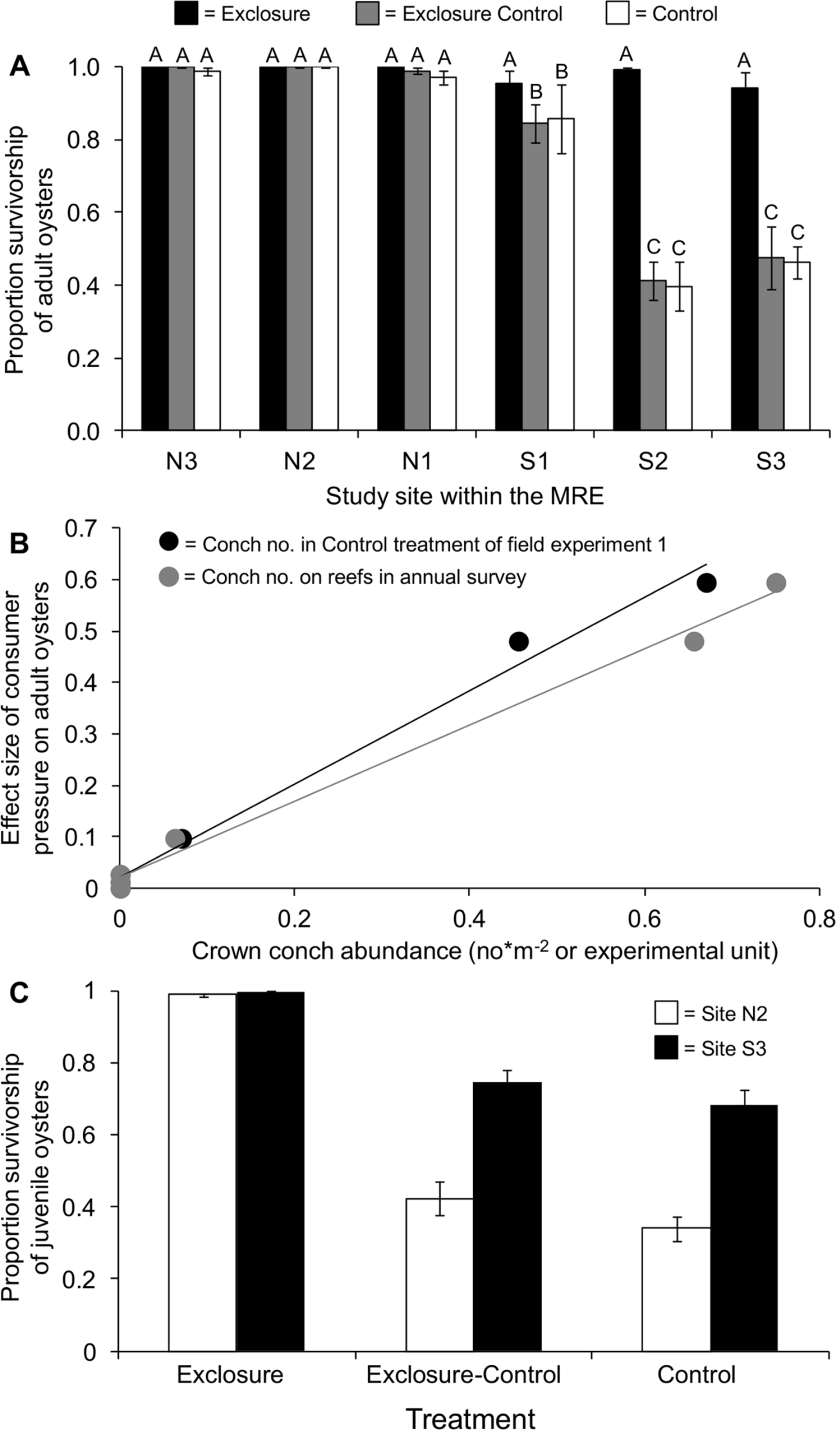
Results from two field experiments in the Matanzas River estuary. In field experiment 1, the (A) mean (SE) survivorship of adult oysters after 2 weeks, and (B) the relationship between the effect size of consumer pressure on adult oysters and crown conch abundance in control treatments (black); the same effect size also plotted as a function of conch abundance observed in annual surveys (gray). In field experiment 2, the (C) mean (SE) survivorship of juvenile oysters after 2 weeks. In (A), closed bars denote exclosure treatments, gray bars denote exclosure controls, and open bars denote control treatments. In (C) open bars denote the northern site and closed bars denote the southern site. For (A) and (C), different letters above bars denote significant differences (p < 0.05) based on Tukey’s post-hoc test.

After 2 weeks, adult oyster survivorship was uniformly high except in the control and exclosure-control treatments of experiment 1 at the two southern sites (Table 3 and Fig 2A). Because survivorship in exclosure treatments at all sites remained high, we attributed low survivorship in the controls to consumer pressure. Furthermore, we observed conchs in the control and exclosure-control treatments at the two southernmost sites. In experiment 1, the strength of consumer pressure was positively correlated with conch abundance (experiment: y = 0.740x + 0.021, R^2^ = 0.99; survey: y = 1.092x – 0.020, R^2^ = 0.99; Fig 2B). Over time, stress or disease could have caused spatial variation in survivorship. But after 2.5 months, oyster survivorship in conch exclosure treatments did not differ spatially (Table 3).

In experiment 2, juvenile survivorship remained high in exclosures and equally low in the control and exclosure-controls (Table 3 and Fig 2C). Survivorship within the latter two treatments was higher at the southern site than at the northern site. Because this short-term, predator-induced mortality of juvenile oysters was negatively correlated with spatial patterns of oyster biomass and mortality index on natural reefs (Fig 1B), which initially motivated our study, patterns in juvenile oyster survivorship were not considered further. Our decision was reinforced when post-settlement survivorship in the exclosure treatment did not differ spatially after an additional three months (Table 3).

### Ultimate causal factor(s)

#### Field-tethering experiment

**Fig 3:**
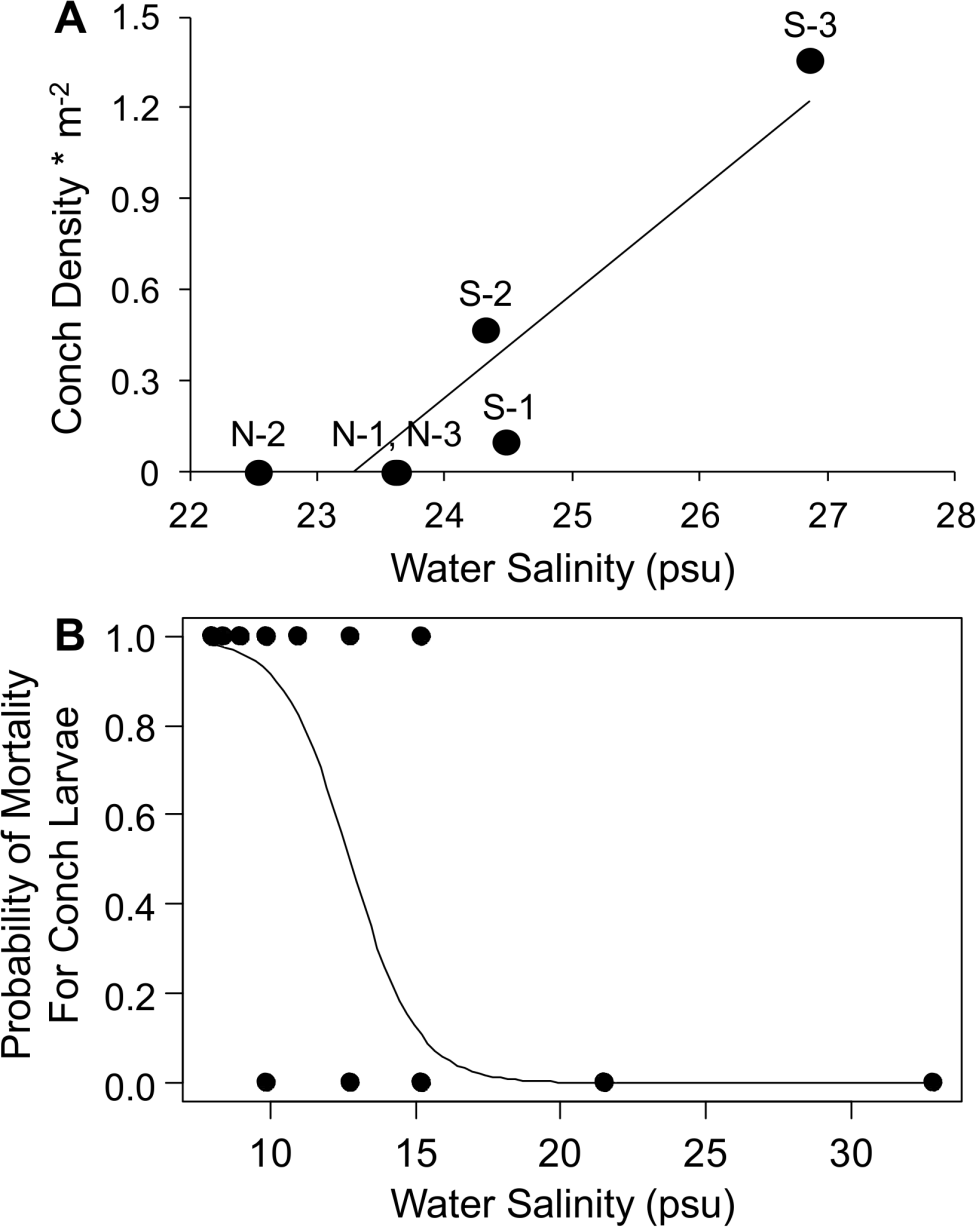
Relationships between crown conchs and water salinity based on observational and experimental evidence. (A) The relationship between crown conch abundance and water salinity of the six study sites in the Matanzas River estuary. (B) In experiment 3, results of logistic regression that examined the probability of larval mortality as a function of salinity after 14 days of exposure.

In experiment 3, conch density differed among sites, with conchs being nearly absent near the Matanzas inlet (site S1) and steadily increasing south of the inlet (Fig 1C). In contrast, survivorship of tethered adult and sub-adult conchs after 3 months did not differ across these sites (F_2,29_ = 0.32, p = 0.73); it also did not differ with conch size (F_2,29_ = 1.02, p = 0.37) or with the interaction between site and size (F_3,24_ = 1.09, p = 0.37). In addition, conch growth did not differ spatially (F_2,12_ = 1.93, p = 0.19). Over 12 months, conch survivorship differed spatially (F_1,16_ = 4.7, p < 0.05), with higher survivorship closer to Matanzas inlet. This result, however, appears relatively unimportant because it was opposite to the observed abundance of conchs. In contrast to the insignificant results of our tethering experiment, our model-selection results suggested spatial variation in salinity as the best predictor of conch abundance (w = 0.64, ∆AIC_c_ = 0.0, S4 Table; y = 0.20x – 4.48, R^2^ = 0.91; Fig 3A). Consequently, we suspected that salinity may influence conch larvae rather than adult and sub-adult conchs.

#### Laboratory experiment

In experiment 4, which was a laboratory experiment conducted by [26], the mortality of conch larvae after 7 days remained zero regardless of salinity. After 14 days, however, salinity level significantly influenced the probability of larval mortality, with mortality increasing when salinity was 15 and below (salinity z-value -3.026, p < 0.01; Fig 3B).

#### Environmental time series of MRE

**Fig 4:**
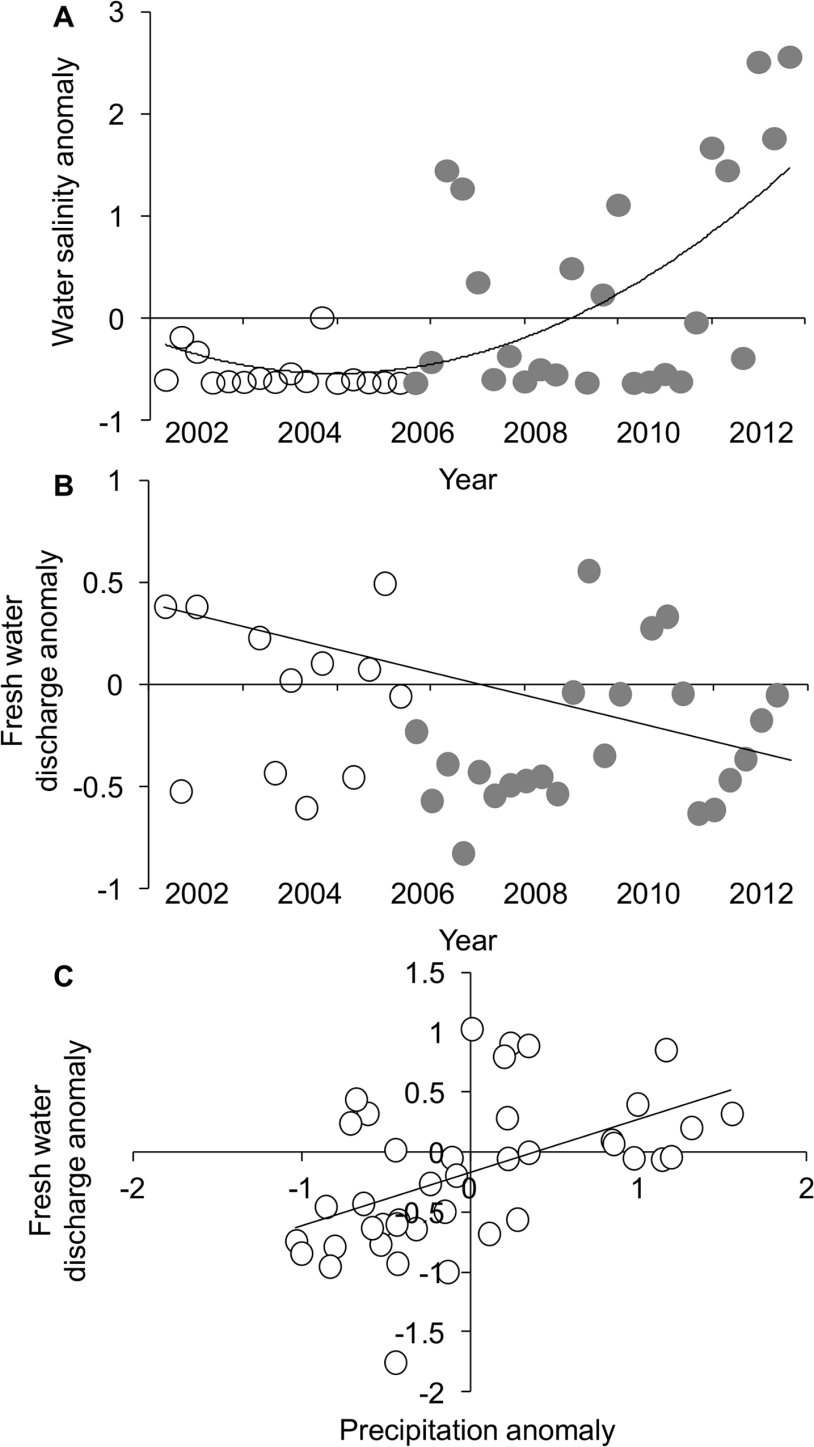
Environmental variation of the Matanzas River estuary. (A) From 2002 to 2011, the seasonal anomaly of water salinity. (B) From 2002 to 2012, the seasonal anomaly of freshwater discharge. (C) The relationship between the seasonal anomaly of freshwater discharge and the seasonal anomaly of local precipitation. In (A-B), open symbols denote data points before 2006, and gray symbols denote data after 2006. All data were normalized according to the overall seasonal mean and seasonal standard deviation ([overall mean—seasonal mean] / overall standard deviation).

According to local stakeholders, conchs first became abundant south of Matanzas inlet in 2006, and oyster reefs declined 2 years later (S1 Table). Paralleling this time frame, seasonal salinity before 2006 mostly remained below the decadal average but increased sharply thereafter (F_1,38_ = 6.52, p = 0.01; y = 0.002x^2^–0.06x – 0.21; R^2^ = 0.41; Fig 4A). While this increase in salinity was not related to local precipitation (F_1,38_ = 1.03, p = 0.32), it was related to the amount of seasonal discharge from Pellicer Creek (F_1,38_ = 6.17, p = 0.02), which has decreased since 2002 (F_1,38_ = 4.75, p = 0.04; R^2^ = 0.11; Fig 4B). Interestingly, we detected a positive correlation between seasonal freshwater discharge and precipitation (F_1,38_ = 8.64, p < 0.01, y = 0.40x + 0.02, R^2^ = 0.19; Fig 4C), suggesting that discharge may be a link between precipitation and the observed increase in seasonal salinity.

### Regional-scale survey

**Fig 5:**
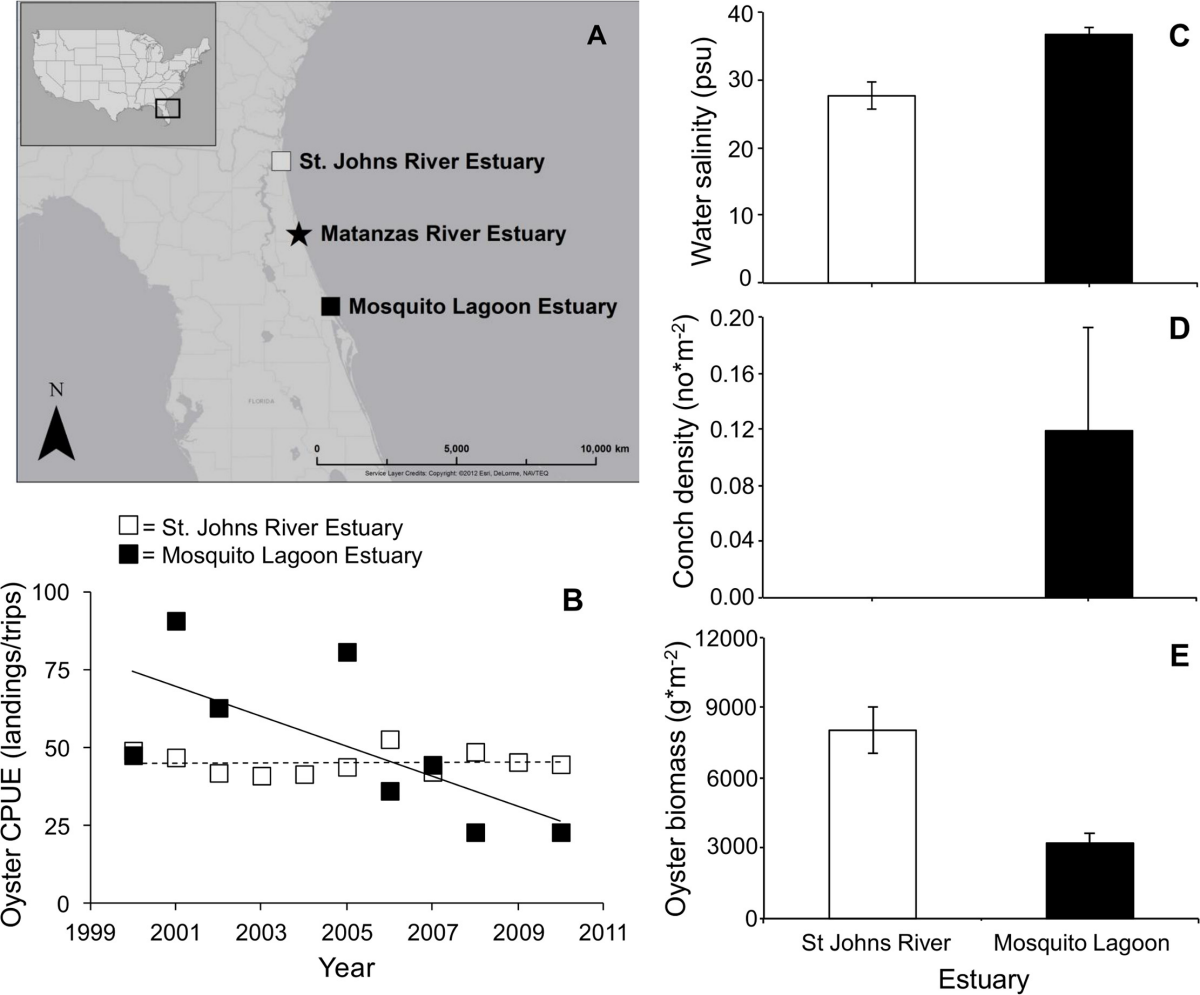
In an estuary north and south of the Matanzas River estuary, variation in the catch per unit effort of oysters, water salinity, crown conchs, and biomass of oyster reefs. (A) Map of the Floridian ecoregion illustrating the location of the Matanzas River Estuary (star symbol) as well as a northern (open square) and southern (closed square) estuary. (B) The annual CPUE of oysters from 2000 to 2011 for the northern and southernmost estuaries. (C) Mean (SE) salinity of water in the northernmost and southernmost estuary. Mean (SE) abundance of conchs (D) and oyster biomass (E) in the northernmost and southernmost estuaries of this ecoregion. Asterisks denote significant differences (p < 0.05). All maps were produced in ArcGIS by E. Pettis.

In the St. Johns estuary, oyster CPUE has changed little over the last decade (y = 0.76x - 1472.2, R² = 0.22; Fig 5A and 5B). In contrast, oyster CPUE in the Mosquito River Lagoon estuary has declined over the last decade (y = -6.25x + 12587, R² = 0.74; Fig 5B). Currently, reef biomass is significantly higher in the St. Johns estuary than in the Mosquito River Lagoon estuary (F_1,47_ = 12.09, p < 0.001; Fig 5E). The Mosquito River Lagoon estuary currently has significantly higher water salinity (F_1,12_ = 16.45, p < 0.002; Fig 5C) and a greater conch density than the St. Johns estuary (Fig 5D). Like water salinity in the MRE, water salinity in the Mosquito River Lagoon estuary has increased over the last decade (y = 0.05x – 1.05; R^2^ = 0.36; F_1,43_ = 24.26, p < 0.001).

## Discussion

This study suggests that oyster reefs in Florida, USA, are experiencing strong consumer pressure because of environmental change in the form of lower freshwater input and higher water salinity. In the MRE, oysters have been commercially harvested north and south of its major inlet since at least 1965 (S1 Table). But more recently, reefs south of the inlet have not supported harvesting, and these reefs now contain less living biomass and greater numbers of recently deceased oysters than reefs north of the inlet (Fig 1B). Although this decline in oyster reefs is positively correlated with the abundance of an oyster consumer (the crown conch) and water salinity, our experiments revealed that oysters persist regardless of salinity as long as conchs are excluded (Fig 2A).

While conchs are the proximal cause of oyster loss, we initially suspected water salinity as the ultimate cause either by decreasing conch growth and survivorship or by controlling the abundance of a predator of conchs—and thus the presence of a trophic cascade. Our tethering experiment demonstrated, however, that conchs persist regardless of the southward distance from the inlet. In the absence of spatial variation in predation, and consequently in the potential for a trophic cascade, we had two additional reasons to suspect that water salinity controls conchs. First, local stakeholders of the MRE estimated that conchs became abnormally abundant in 2006 (S1 Table), a time period that coincides with an anomalous increase in water salinity (Fig 4A). Second, across the MRE, conch abundance and water salinity are positively correlated (Fig 3A). Nevertheless, these two findings were contradicted by our tethering experiment, which showed that lower salinity did not influence the survivorship of tethered *adult* or *sub-adult* conchs. But this contradiction is reconciled by a previously published experiment, which showed that the mortality of conch *larvae* increases at water salinities ≤ 15 psu (Fig 3B; [26]). Given this mechanistic link between low salinity and larval mortality of the crown conch as well as the spatial and temporal associations between salinity and the crown conch, we conclude that salinity is the ultimate cause of oyster losses in the MRE.

If salinity controls conchs, then resource managers should understand the factors that control salinity. A number of factors may underlie the salinization of the MRE including a declining tidal prism of its major inlet, which has narrowed by ~30% since 2006; a narrower tidal prism exchanges less water between the ocean and the estuary, leading to longer water residence times, more evaporation, and consequently elevated water salinity [34]. Because sites with conchs are not located near the inlet, however, their salinity is primarily influenced by freshwater discharge, evaporation, and precipitation [27]. Although we lacked data on evaporation, we found that salinity was significantly associated with freshwater discharge but not with precipitation. The lack of statistical significance for precipitation was most likely due to intense precipitation events such as that of spring 2009, which may have strong, transient effects on salinity. Interestingly, freshwater discharge was positively associated with local precipitation, suggesting that precipitation’s influence on salinity may be mediated through freshwater discharge.

In support of this suggestion, 35% of the reduction in discharge from Pellicer Creek can be attributed to a decline in precipitation throughout the creek’s watershed [35]. Like most watersheds in northern Florida (S1 Fig), Pellicer Creek’s is relatively small. Consequently, precipitation likely accounts for greater than 35% of the reduction in discharge from Pellicer Creek. Given that this system has experienced severe drought [21], we hypothesize that the salinization of the MRE is due to reduced freshwater discharge (Fig 4A and 4B) resulting from reduced precipitation.

Since the Mesozoic, oysters have relied on the lower salinity of estuaries for protection from marine predators and disease [36]. However, our study illustrates that these estuarine niches are highly sensitive to lulls in freshwater discharge, which can increase salinity and rapidly promote a consumer outbreak. With climate change, the consequences of prolonged lulls in precipitation and freshwater discharge may be especially pertinent for estuaries in the Floridian ecoregion, because they already receive the lowest amount of freshwater discharge among estuaries on the Atlantic coast (S1 Fig). The results presented here indicate that a salinity-induced increase in conchs and a resultant decrease in oysters may be occurring throughout a large portion of this ecoregion (Fig 5). A similar mechanism may also be affecting the N. Gulf of Mexico ecoregion, which is the leading producer of commercial oyster landings [16]. In an important estuary of this ecoregion, a recent collapse of the oyster fishery coincided with elevated water salinity [37]. Oyster reefs of this ecoregion are predominately subtidal, and researchers have hypothesized that a different gastropod (the southern oyster drill, *Stramonita haemastoma*) increases its consumption of oysters with increasing water salinity [25,38]. Even if oysters avoid predation, they must still contend with an increasing incidence and intensity of disease (*Perkinsus marinus*) under elevated water salinity [22,23,39]. Because these ecoregions are purportedly two of the few remaining areas of the globe with viable oyster populations [16], conserving some of the last remaining oysters depends on accounting for the effects of precipitation and salinity on gastropod predators, disease, and oysters.

In most systems, the depletion of resources by consumers eventually causes the consumer population to crash, which in turn allows resources to recover. Examples of consumer–resource oscillations include those generated by wolves and moose [40], lynx and hare [41], as well as sea urchins and seagrass [42]. Given the apparently general nature of this consumer–resource dynamic, why has conch abundance remained relatively high even as oysters decline? We offer two, non-mutually exclusive hypotheses. First, in addition to consuming oysters, the crown conch consumes clams, other crown conchs, and decomposing fishes and decomposing invertebrates [18,43]. Thus, a generalist diet may promote population stability at high densities even when local oyster reefs are declining. Second, our unpublished experiments and the observation that oysters are much smaller on reefs with conchs suggest that conchs selectively consume large oysters (i.e., shell length > 35 mm) and/or that the chemical cues of the crown conch cause oysters to allocate energy toward shell thickening as opposed to growth [44]. Because oysters smaller than 35 mm are reproductively viable, reef productivity south of the Matanzas inlet may provide conchs with adult oysters in the short term. Collectively, these un-tested hypotheses indicate how resources may still be unlimited for a large population of conchs.

Will large conch populations expand northward? Because conchs are direct developers with crawl-away juveniles, they are expected to have little potential for range expansion. This expectation, however, is inconsistent with the 90-km northward expansion of conchs in the last 50 years and their genetic similarity along the Florida coastline [28]. In addition to rafting on vegetation, conchs may be dispersed by anthropogenic activities. This range shift to the north, however, may stall at the MRE because freshwater discharge into estuaries increases northward (S1 Fig), and the increased volume may buffer against salinization. Another limitation to further northward expansion concerns the geomorphology of the coastline. At more northern sites, tidal amplitudes are higher [45], and low tides periodically overlap with cold air temperatures in winter, resulting in high conch mortality [46]. Therefore, future research should evaluate the individual and combined influences of air temperature and water salinity on the range expansion of conchs.

If salinization subsides, will MRE oyster reefs recover? The life history of the eastern oyster provides optimism, because oyster larvae are planktonic and have the potential to rapidly colonize relatively distant locations. If a nearby and viable source population exports larvae to the southern portion of the MRE, then viable oyster reefs may stabilize the sink populations of southern reefs [47]. However, the potential for this source–sink dynamic in the Floridian ecoregion likely decreases with an increasing duration of high salinity and consumer pressure. In this ecoregion, riverine inputs are high in sediment [48], which normally helps reefs persist by burying the dead, structural base of the reef and protecting it from taphonomical erosion. But for reefs to persist, the rate of reef burial must be balanced by an equal rate of oyster production on the reef surface via recruitment, growth, and survivorship [49]. As consumer pressure and sediment loading continue, an imbalance may develop such that there is no longer a reef surface available to larvae.

It is important to recognize that at modest densities, crown conchs help maintain salt marshes and possibly oyster reefs. In salt marshes, conchs cause periwinkle snails (*Littoraria irrorata*) to spend more energy hiding rather than overgrazing an important plant (*S*. *alterniflora*) [50]. On oyster reefs, conchs pry open oyster valves in order to consume internal tissue, which leaves behind a relatively clean internal cavity within the oyster shell. Because oyster larvae settle within these cavities, normal conch abundances may promote oyster recruitment. In contrast, large conch populations can decimate oyster reefs. Determining whether the conch–oyster dynamic in our study system represents a permanent decline (i.e., new steady state) will require further research. In particular, regional-scale surveys, experiments, and restoration should be repeatedly conducted in areas with and without conchs, and these results should be integrated with model simulations of system dynamics before and after the onset of a conch outbreak [51]. Without a deeper understanding of this conch–oyster dynamic, oyster conservation and restoration efforts throughout this ecoregion may be ineffective.

## Supporting Information

S1 Dataset Data used in statistical analyses for results of Figs 1–5. (XLSX)

S1 Table Stakeholder estimates of (a) when crown conchs became abnormally abundant in the southern portion of the MRE and (b) when oyster reefs of the southern MRE began to decline. (DOCX)

S2 Table Model selection results for predictors of spatial variation in oyster biomass in the MRE. (DOCX)

S3 Table Model selection results for predictors of spatial variation in adult oyster mortality index in the MRE. (DOCX)

S4 Table Model selection results for predictors of spatial variation in crown conch abundance in the MRE. (DOCX)

S1 Fig Fraction of land area draining to Atlantic coastline from north to south. (PPTX)
