# Supplementary material for: Drought Increases Consumer Pressure on Oyster Reefs in Florida, USA
Source: PLoS One. 2015 Aug 14;10(8):e0125095. doi: 10.1371/journal.pone.0125095 (PMC4537192; doi:10.1371/journal.pone.0125095)
Supplement: S1 Table — (DOCX) [file pone.0125095.s002.docx]

**S2 Table.** List of local stakeholders in the Matanzas River Estuary (MRE) and research scientists with first-hand knowledge of (a) when crown conchs became abnormally abundant in the southern portion of the MRE and (b) when oyster reefs in the southern portion of the MRE began to decline. Dates are rounded to the year. Grey text represents the inferred date of conch outbreak and initial decline of reefs according to the paired estimate. When possible, we have indicated the years of experience for each stakeholder in the second column of the table.

| **Data Source** | **First began**  **work in the MRE** | **Estimated year of conch**  **outbreak** | **Estimated year in which the MRE last lacked conchs** | **Estimated year of**  **decline in oyster reefs** | **Estimated last year when reefs were healthy** |
| --- | --- | --- | --- | --- | --- |
| GTM NERR personnel 1 |  | >2002 | 2001 |  |  |
| University of Florida Whitney Marine Lab  Scientist 1 | 1994 | >2005 | 2004 | >2005 | 2004 |
| University of Florida Whitney Marine Lab  Scientist 2 | 1985 | 2009 | 2008 |  |  |
| Karl and Hayes. 2012. *J. Heredity* | 1999 | >2004 | 2003 |  |  |
| Shell curator | 1975 | >2009 | 2008 |  |  |
| St. Johns River Water Management District |  |  |  | 2008 | <2007 |
| Commercial Fisherman 1 | 1970 | 2004 | 2003 |  |  |
| Commercial Fisherman 2 | 1965 | >2005 | 2004 | 2008 |  |
| Ecotourism business owner | 2006 | 2008 | 2007 | 2011 | 2010 |
| First media report |  |  |  | 2010 | <2009 |
| **Mean** |  | **2006** | **2005** | **2008** | **2007** |
