# Supplementary material for: Drought Increases Consumer Pressure on Oyster Reefs in Florida, USA
Source: PLoS One. 2015 Aug 14;10(8):e0125095. doi: 10.1371/journal.pone.0125095 (PMC4537192; doi:10.1371/journal.pone.0125095)
Supplement: S3 Table — (DOCX) [file pone.0125095.s004.docx]

**S4_Table.** Summary of model-selection results for the mortality index of adult oysters at study sites in the Matanzas River estuary as a function of measured biological or environmental variables. Fitted parameters include (a) null model (the only factor is an intercept value of 1), (b) crown conch abundance (number per m^2^; average value of four reefs per site), (c) salinity (psu; average of monthly medians), (d) dissolved oxygen concentration (mg/L; average of monthly samples), (e) proportion of time that the reef is inundated (average of 2-week medians), (f) water temperature (°C; average of monthly medians), (g) chlorophyll *a* concentration in the water column (μ/L; average of monthly samples), and (h) mud crab abundance (number per m^2^; average value of four reefs per site). AIC difference (∆AIC*_c_*) is the difference between the AIC*_c_* of model *i* and the lowest AIC*_c_* observed. Akaike weight (*w_i_*) is calculated as the model likelihood, exp(-∆*_i_* / 2), normalized by the sum of all model likelihoods; values close to 1 indicate greater confidence in the selection of a model.

| **Model** | **df** | **(∆AIC*_c_*)** | ***w_i_*** |
| --- | --- | --- | --- |
| Null | 3 | 10.7 | 0.004 |
| **Conch abundance** | **4** | **0.0** | **0.98** |
| Salinity | 4 | 10.6 | 0.005 |
| Dissolved oxygen | 4 | 18.0 | < 0.001 |
| Time reef inundated | 4 | 13.7 | 0.001 |
| Water temperature | 4 | 17.0 | < 0.001 |
| Chlorophyll *a* | 4 | 20.7 | <0.001 |
| Mud crab abundance | 4 | 17.8 | <0.001 |

∆AIC*_w_* values < 4 indicate plausible hypotheses.

The model in bold type represents the most explanatory variable.
