# Supplementary material for: Drought Increases Consumer Pressure on Oyster Reefs in Florida, USA
Source: PLoS One. 2015 Aug 14;10(8):e0125095. doi: 10.1371/journal.pone.0125095 (PMC4537192; doi:10.1371/journal.pone.0125095)
Supplement: S4 Table — (DOCX) [file pone.0125095.s005.docx]

**S5_Table.** Summary of model-selection results for crown conch abundance in the Matanzas River estuary. Fitted parameters include (a) null model (the only factor is an intercept value of 1), (b) water temperature (°C; average of monthly medians), (c) water salinity (psu; average of monthly medians), (d) dissolved oxygen concentration (mg/L; average of monthly samples), (e) chlorophyll *a* concentration in the water column, (f) proportion of time that the reef is inundated (average of 2-week medians), and (g) mud crab abundance. AIC difference (∆AIC*_c_*) is the difference between the AIC*_c_* of model *i* and the lowest AIC*_c_* observed. Akaike weight (*w_i_*) is calculated as the model likelihood, exp(-∆*_i_* / 2), normalized by the sum of all model likelihoods; values close to 1 indicate greater confidence in the selection of a model.

| Model | df | (∆AIC*_c_*) | *w_i_* |
| --- | --- | --- | --- |
| Null | 2 | 1.4 | 0.31 |
| Water temperature | 3 | 9.9 | 0.004 |
| **Salinity** | **3** | **0.0** | **0.64** |
| Dissolved oxygen | 3 | 11.4 | 0.002 |
| Chlorophyll *a* | 3 | 11.2 | 0.002 |
| Time reef inundated | 3 | 10.8 | 0.003 |
| Mud crab abundance | 3 | 6.2 | 0.02 |

∆AIC*_w_* values < 4 indicate plausible hypotheses.

The model in bold type represents the most explanatory variable.
