# Supplementary material for: Drought Increases Consumer Pressure on Oyster Reefs in Florida, USA
Source: PLoS One. 2015 Aug 14;10(8):e0125095. doi: 10.1371/journal.pone.0125095 (PMC4537192; doi:10.1371/journal.pone.0125095)
Supplement: S1 Fig — (PPTX) [file pone.0125095.s006.pptx]

## Slide 1
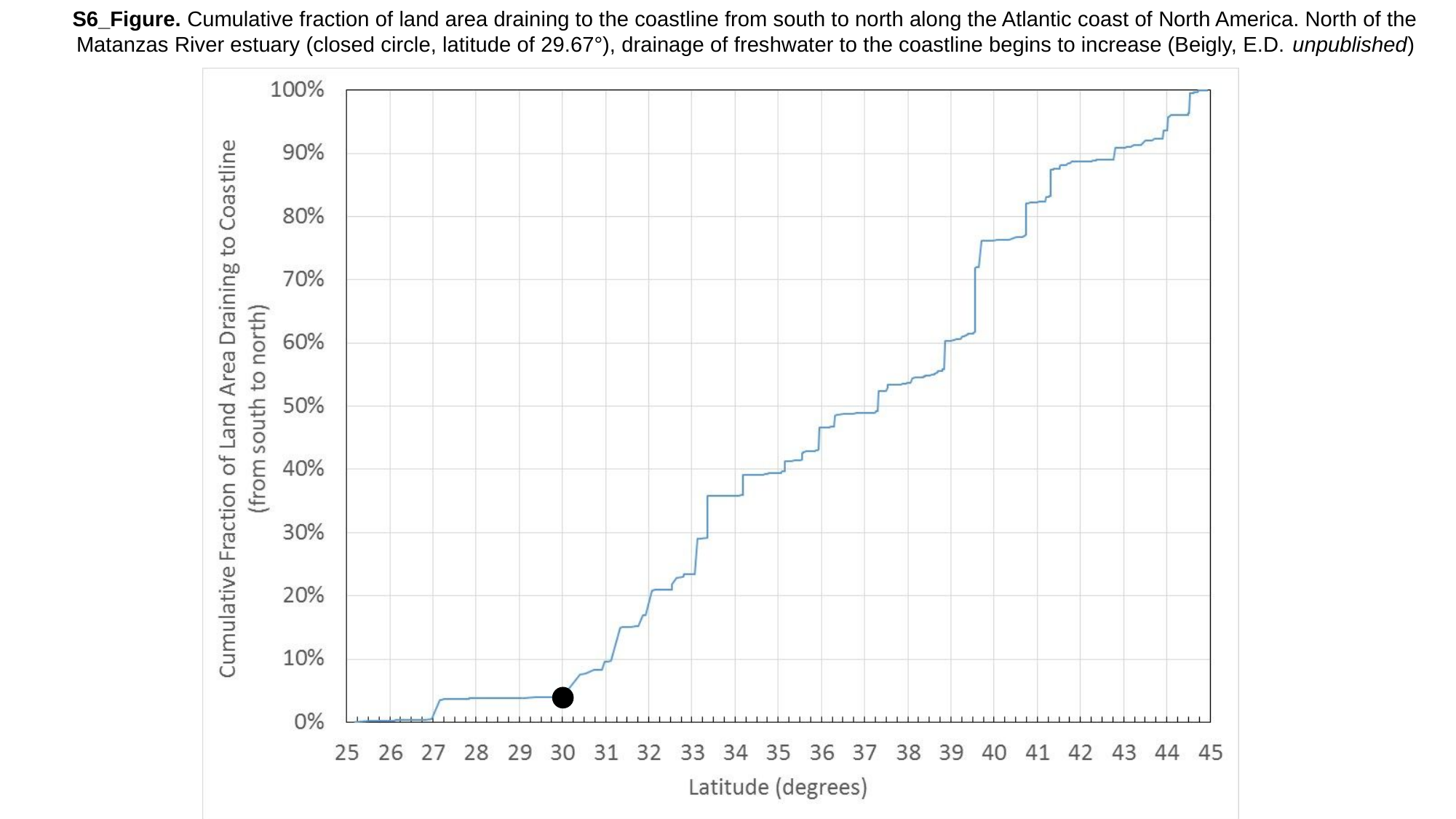

S6_Figure. Cumulative fraction of land area draining to the coastline from south to north along the Atlantic coast of North America. North of the Matanzas River estuary (closed circle, latitude of 29.67°), drainage of freshwater to the coastline begins to increase (Beigly, E.D. unpublished)
